# Insights into the Mechanism of Graphene Acting on Water and Chloride Ion Permeability of Cement-Based Materials

**DOI:** 10.3390/ma16103881

**Published:** 2023-05-22

**Authors:** Jianmiao Dong, Jiaqiao Zhuang, Wanjin Li, Mingxuan Zou, Qi He, Shuqiong Luo

**Affiliations:** 1 School of Civil Engineering and Architecture, Guangxi University of Science and Technology, Liuzhou 545006, China; 2 College of Biological & Environmental Sciences, Zhejiang Wanli University, Ningbo 315000, China; 3 Henan Key Laboratory of Materials on Deep-Earth Engineering, School of Materials Science and Engineering, Henan Polytechnic University, Jiaozuo 454003, China

**Keywords:** graphene, cement-based materials, permeability, microstructure, mechanism

## Abstract

Due to its excellent mechanical properties and high aspect ratio, graphene can significantly improve the water and chloride ion permeability resistance of cementitious materials. However, few studies have investigated the effect of graphene size on the water and chloride ion permeability resistance of cementitious materials. The main issues are as follows: How do different sizes of graphene affect the water and chloride ion permeability resistance of cement-based materials, and by what means do they affect these properties? To address these issues, in this paper, two different sizes of graphene were used to prepare graphene dispersion, which was then mixed with cement to make graphene-reinforced cement-based materials. The permeability and microstructure of samples were investigated. Results show that the addition of graphene effectively improved both the water and chloride ion permeability resistance of cement-based materials significantly. The SEM (scanning electron microscope) images and XRD (X-ray diffraction) analysis show that the introduction of either type of graphene could effectively regulate the crystal size and morphology of hydration products and reduce the crystal size and the number of needle-like and rod-like hydration products. The main types of hydrated products are calcium hydroxide, ettringite, etc. The template effect of large-size graphene was more obvious, and a large number of regular flower-like cluster hydration products were formed, which made the structure of cement paste more compact and thus significantly improved the resistance to the penetration of water and chloride ions into the matrix of the concrete.

## 1. Introduction

Cement-based materials are the most widely used materials for construction projects. With the rapid development of the economy and the vigorous progression of urbanization, higher requirements are presented for cement-based materials, such as their mechanical performance and durability [1,2,3,4,5]. The hydration process of cement plays an important role in dictating the performance of cement-based materials. A large number of microscopic cracks and pores are produced in cement-based materials during hydration, resulting in defects including high brittleness, poor corrosion resistance, and easy cracking [6,7,8,9]. The introduction of fiber reinforcement and/or nanoparticles into cement composites is a common modification method, but fiber and ordinary nanomaterials cannot control the microstructure of the cement hydration products, and it is difficult to block harmful substances from penetration and to protect the reinforcement from corrosion. As a result, reinforcement corrosion may occur [10,11,12]. Permeability is one of the most important indexes of the durability of cement-based materials. Research on the permeability resistance of cement-based composites is of great practical significance to ensure the safety of building structures and also save maintenance costs.

Graphene, as the thinnest two-dimensional nanomaterial known in the world, boasts a two-dimensional network lattice structure formed by single-layer carbon atoms with a sp2 hybridized orbital. It has excellent mechanical, thermal, electrical, and barrier properties and a large specific surface area [13,14,15,16,17]. Previous studies have shown that graphene, thanks to its filling, template, bridging, and drawing effects, can regulate the growth of hydration products and significantly improve the mechanical properties and durability of cement-based materials [6,18,19]. Lavagna et al. [20] added graphene with a dosage of 0.1% by weight and an oxygen content in graphene of 0~45%, respectively, into cement-based materials. It was found that when the oxygen content of graphene was 5% by weight, the mechanical properties of cement-based materials were the best, and both strength (flexural and compression) and toughness were significantly improved with respect to pure cement (80% increase in flexural strength, 30% in compression strength, 20% in toughness). Mohammed et al. [21] and Li et al. [22] reported that graphene oxide can reduce the porosity and refine the pore size of cement-based materials so that the cement composites can achieve the expected water permeability and corrosion resistance. Wang et al. [23] prepared graphene cement paste composites with good dispersion performance by using CO890 as a dispersant and studied the chloride ion permeability resistance of graphene cement-based composites. The experimental results show that the addition of graphene could effectively reduce the chloride ion permeability depth and migration coefficient of cement paste. Du et al. [24] conducted an experimental study on the transmission characteristics of graphene nanosheets (GNPs) mixed with cement concrete under the action of chloride and water. The results show that the porosity and mobility of concrete containing 1.5% GNPs were reduced by the most, and the permeability depth, chloride ion diffusion, and migration coefficient were reduced by 64%, 70%, and 31%, respectively, compared with the control samples without GNPs. Tong et al. [25,26,27] studied the effects of graphene on the freeze-thaw resistance of cement mortar. Their results show that different types of graphene could improve the freeze-thaw resistance of cement mortars.

Existing studies have shown that there are generally two methods for using graphene or graphene oxide to improve the durability of cement-based materials. One is to add graphene or graphene oxide to cement-based materials as an admixture, and the other is to combine it with other substances to prepare nanocomposite materials as coatings on the surface of cement-based materials. So far, research has been focused on the preparation methods of graphene and the effects of the oxygen content of graphene on improving the durability of cement-based materials. Very few researchers have explored the influence of graphene on the durability of cement-based materials from the perspective of its size. The existing research generally believes that graphene can regulate the growth form of cement hydration products and has a template effect [28,29]. For example, LV et al. [30,31] found that GO with an oxygen content of 18.65% and 25.53% by weight can make cement hydration products into flower-like microcrystals, but the template effect of graphene and the morphology of cement hydration products on the surface of graphene have not been directly observed.

Based on previous research, the effects of graphene with different sizes on the water and chloride ion permeabilities of cement-based materials were studied in this research by the water permeability height method and the rapid chloride ion migration method, respectively. Combined with XRD, SEM, EDS (energy-dispersive X-ray spectroscopy) and other characterisation methods, the changes in hydration product morphology and hydration rate in cement-based materials with graphene and graphene oxide were analyzed. To observe the interaction between graphene and cement-based materials more intuitively, cement paste/graphene/cement particle samples were prepared by a new method. The sample prepared by the new method allows cement to directly adhere to the surface of graphene instead of simply mixing graphene with cement. Therefore, the growth morphology and aggregation mode of cement hydration products on the graphene surface can be clearly observed by SEM, and the mechanism of graphene improving both water and chloride ion permeability resistance was studied. 

## 2. Experimental

### 2.1. Materials

Cement: P.II42.5 Portland cement produced by Guangxi Yufeng Group Co., Ltd. (Liuzhou, China), conforming to Chinese Standard GB175-2007, was used for preparing the samples. The main properties of the cement used are shown in Table 1.

Polycarboxylic acid water reducers (PCs), produced by Fuclear Technology Suzhou Co., Ltd. (Suzhou, China), with a water reduction efficiency of 30%, were used as a superplasticizer for preparing cement samples.

Graphene dispersion: Two types of peeling graphene (PG1 and PG2) slurry prepared by Guangxi Qinglu Technology Company (Liuzhou, China) were used for preparing graphene-reinforced cement composites in this study. The solid contents of the two types of graphene dispersion were 5% and 4% by weight, respectively. The graphene in the dispersion was in the agglomeration state as it was supplied. After treatment, a graphene slurry was prepared.

Standard sand: the standard sand used for making cement mortars in this study was the ISO standard sand produced by Xiamen ISO Standard Sand Co., Ltd. (Xiamen, China)

Water: distilled water was used for all cement samples.

### 2.2. Preparation of Graphene Dispersion and Its Dispersion Test

According to previous findings [19], the best preparation method for PG dispersion was as follows: the PG slurry and PCs required by each group of test pieces with distilled water were mixed and then stirred evenly with a glass rod (the ratio of PG, PCs, and distilled water satisfies the mix proportion requirements in Table 2 and Table 3) to form a diluted PG/PCs mixture, which was dispersed by mechanical stirring, followed by ultrasonic dispersion for 15 min to form PG dispersion.

**Figure 1:**
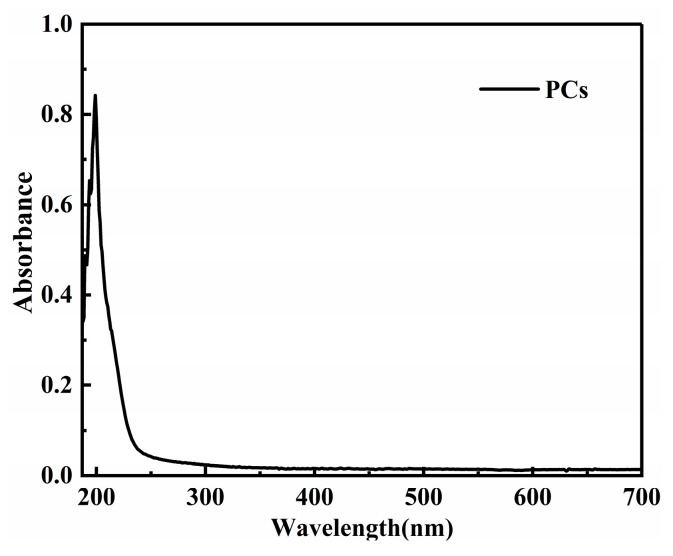
Spectrophotometric curve of the PCs solution.

**Figure 2:**
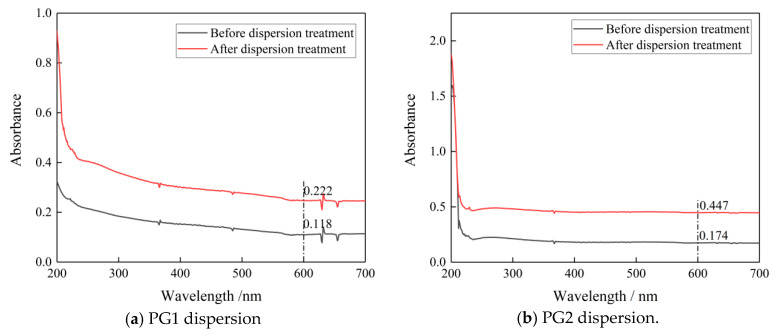
Spectrophotometric curves of the two graphene dispersions before and after dispersion treatment.

To observe the dispersion effect, UV-vis spectrophotometry tests were carried out on the two groups of PG dispersion before and after mechanical stirring and ultrasonic dispersion treatment. The mechanical stirring and ultrasonic dispersion processes together lasted 15 min and the test wavelength was 187~700 nm. To eliminate the influence of PCs in the dispersion on the absorbance of graphene dispersion, the spectrophotometry test was carried out on PC solutions with the same concentration. Results show that the absorbance of the PC solution tended to approach zero after 600 nm (as shown in Figure 1). Therefore, the wavelength of 600 nm was selected for analysis in this paper. The spectrophotometric curves of PG1 and PG2 graphene dispersions are shown in Figure 2. 

According to Figure 2a,b, at the wavelength of 600 nm, the absorbance of PG1 dispersion before dispersion treatment was 0.118, and that after dispersion treatment reached 0.222, which is 88.1% higher. The absorbance of PG2 dispersion before dispersion treatment was 0.174, and after dispersion treatment, it reached 0.447, which is 163% higher than. It can be seen that the dispersion degrees of PG1 and PG2 were significantly improved after mechanical stirring and ultrasonic dispersion treatment for 15 min. After the PG1 and PG2 dispersions were left standing for 2 h, it was found that the PG dispersions had no obvious stratification, and the liquid was turbid through the observation of the Tyndall light test. It can be seen that the PG dispersions prepared in this test were evenly dispersed and had good stability [29].

### 2.3. Characterization of the Structure and Properties of Graphene

**Figure 3:**
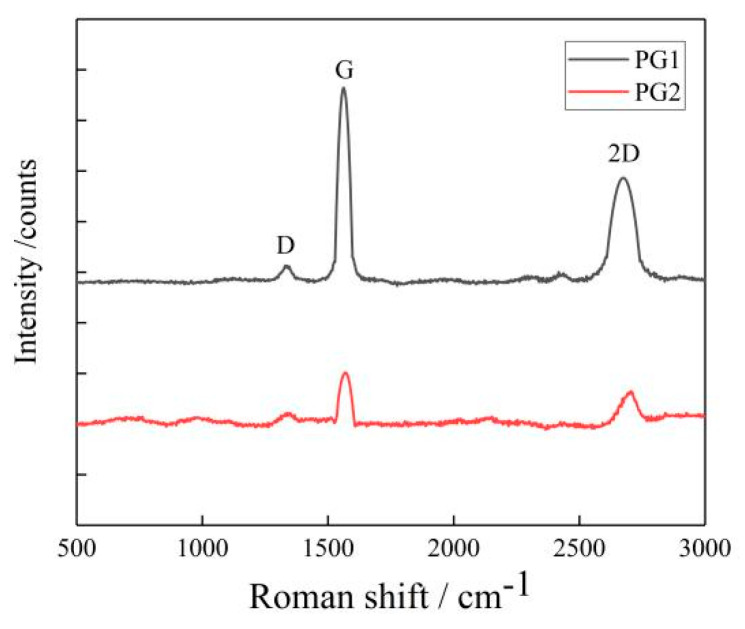
Raman spectrum of graphene.

(1) Two types of graphene were characterized with the XploRA plus Raman spectrometer with a scanning range of 500~3500 cm^−1^, and the number of graphene layers was calculated. The results are shown in Figure 3.

The average number of layers of the two types of graphene can be calculated by the empirical Formula (1) based on their Raman spectrum shown in Figure 3. The average numbers of layers of PG1 and PG2 were determined as 18 and 9, respectively, in this study.

(1) IGI2D=0.14+n10

Of which: *I_G_* represents graphene peak G;

*I*_2*D*_ represents graphene peak 2D, and

n represents graphene layers.

*I_G_*/*I*_2*D*_ indicates the structural and crystalline integrity of graphene, the higher the value of *I_G_*/*I*_2*D*_, the more defects the graphene will have; otherwise, the more complete the structure of graphene, the better the crystallinity. In Figure 1, the *I_G_*/*I*_2*D*_ values of PG1 and PG2 are 0.13 and 0.57, respectively, indicating that PG1 has fewer defects and higher crystallinity than PG2.

**Figure 4:**
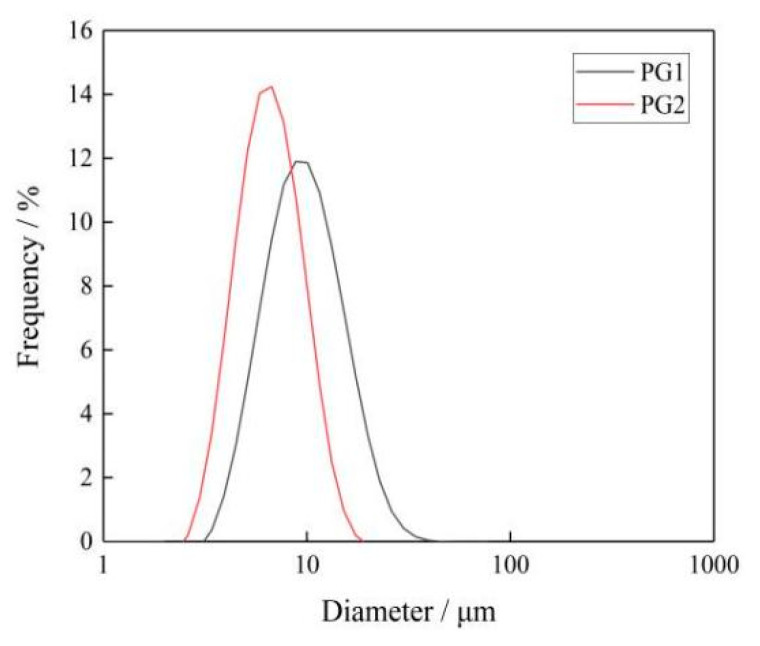
Particle size distribution of graphene.

The sheet diameter of graphene was measured by an LA-960 particle size analyzer [32]. Figure 4 shows the sheet diameter distribution of PG1 and PG2. Since graphene is a sheet structure with irregular shape and size, the mean diameter of the area was selected as the sheet diameter of graphene, and the mean diameter of the area of PG1 and PG2 can be calculated by the particle size analyzer to make it clear to readers. It can be seen from the figure that the sheet diameter of PG1 is 3~40 μm, with an area average of 12.2 μm; while the sheet diameter of PG2 is 2~20 μm, with an area average of 5.6 μm. The characterization results indicate that the sheet diameter of PG1 is greater than that of PG2.

**Figure 5:**
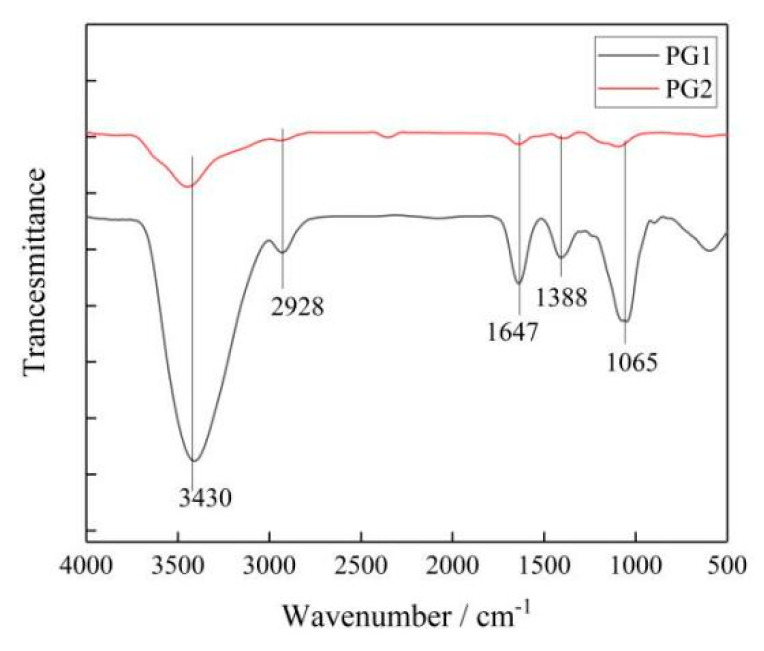
Infrared spectrum of graphene.

(2) The chemical bonds and types of functional groups were measured with a PerkinElmer Frontier FTIR spectrometer. The results are shown in Figure 5.

It can be seen that the two types of graphene had similar absorption peaks, in which 3430 cm^−1^ corresponded to the -OH vibration absorption peak, 2928 cm^−1^ to the C-H vibration absorption peak, 1647 cm^−1^ to the C=C absorption peak, 1388 cm^−1^ to the HBrinterference peak, and 1065 cm^−1^ to the C-O-C stretching vibration absorption peak.

**Figure 6:**
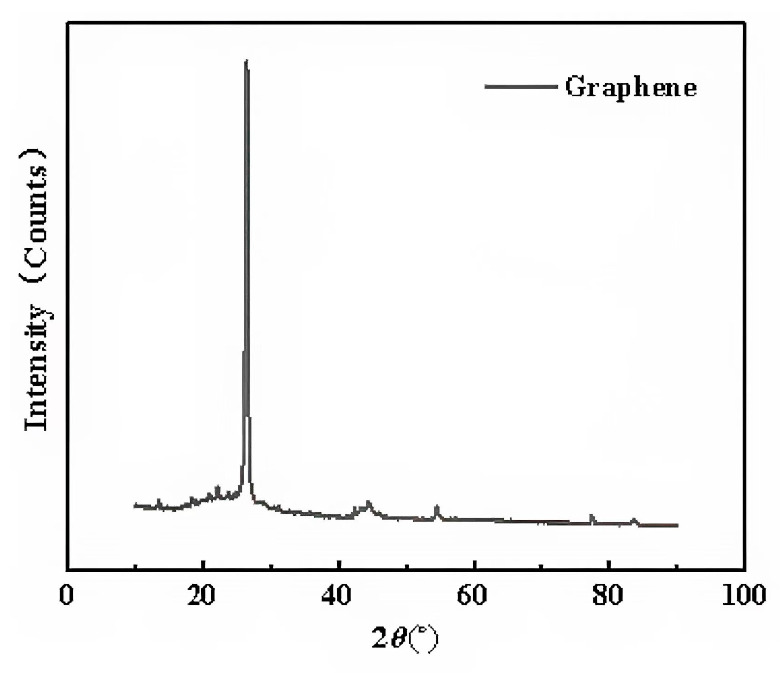
X-ray diffraction pattern of graphene.

The XRD curves of the two types of graphene were measured by D8AA25 XRD. Since the XRD curves of the two types of graphene were almost identical, only one curve is presented, as shown in Figure 6. 

It can be seen that an obvious characteristic carbon peak appeared at 26.2° (2θ). According to the Bragg Equation (2), the layer spacing of the two types of graphene can be obtained.

(2) 2dsinθ=nλ

Of which *d* represents the crystal spacing;

θ represents the X-ray diffraction angle;

*n* represents the reflection series, taking 1 in this study; and

λ represents the wavelength of the incoming rays, which can be 0.154056 nm.

According to Equation (2), the layer spacing of the two types of graphene can be obtained at 0.314 nm.

**Figure 7:**
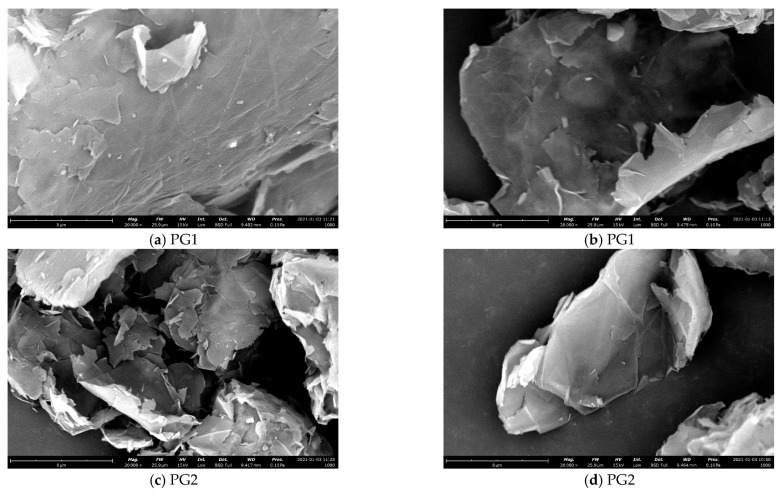
SEM images of the two types of graphene.

(3) The micromorphology of graphene was observed by the Phenom XL G2 scanning electron microscope. Figure 7 shows SEM images of the two types of graphene.

From Figure 7, it can be seen that both PG1 and PG2 exhibit typically two-dimensional sheet structures. PG1 has a larger sheet diameter and fewer surface defects than PG2. According to the EDS analysis, the types and contents of elements in PG1 and PG2 are shown in Table 2. As can be seen from the table, the oxygen content of PG1 is 13.4%, which is higher than that of PG2. Therefore, the degree of oxidation of PG1 is higher than that of PG2, which is consistent with the results characterized by FTIR.

### 2.4. Test Method and Mix Ratio Design

#### 2.4.1. Test Method

(1) A water permeability test was conducted according to the water permeability height method recommended by Chinese Standard GB/T 50082-2009 Standard Test Method for Long-term Performance and Durability of Ordinary Concrete.
(2) The chloride ion permeability test was carried out following the RCM method recommended by Chinese Standard GB/T 50082-2009 Standard Test Method for Long-term Performance and Durability of Ordinary Concrete.

#### 2.4.2. Design of Test Mix Ratio

According to the authors’ previous research results, the content of graphene in the cement paste is 0.02% by weight of cement, while the content of PCs is 0.1% by weight of cement. The mix proportion of cement samples for the water permeability test is shown in Table 2, where W0-, W1-, and W2-series represent cement samples without PG and with PG1 and PG2, respectively. Each sample contains 3350 g of cement, 5025 g of standard sand, and 1139 g of water. 

The mix proportion of cement samples for the chloride ion permeability test is shown in Table 4, where C0-, C1-, and C2-series represent the samples without PG, and with PG1 and PG2, respectively, for the chloride ion permeability test. Each sample consists of 1726 g of cement, 500.54 g of water, and 1.716 g of PCs. 

## 3. Results and Discussion

### 3.1. Effect of Graphene on Water and Chloride Permeability of Cement Pastes

#### 3.1.1. Water Permeability Test

The sample for the permeability test is in a round platform shape with an upper diameter of 175 mm, a lower diameter of 185 mm, and a height of 150 mm. After the samples were removed from their molds after 24 h of initial curing, the excessive cement slurry was brushed away from both ends and tops of the samples with a steel wire brush, and then the samples were cured in the standard maintenance room for 28 d. The samples were sealed in the permeability test mold before the test. The sealing process was as follows: First, the sealing material made of paraffin and rosin at a 5:1 ratio by weight was put into the oven to melt, followed by being daubed with a layer of 1–2 mm thick on the side of the samples with a brush. Afterwards, the samples were pressed into the preheated test molds at a slow speed until the sample reached the bottom of the test mold. The temperature of the test mold was determined just to be able to melt the sealing material slowly but not cause it to flow.

**Figure 8:**
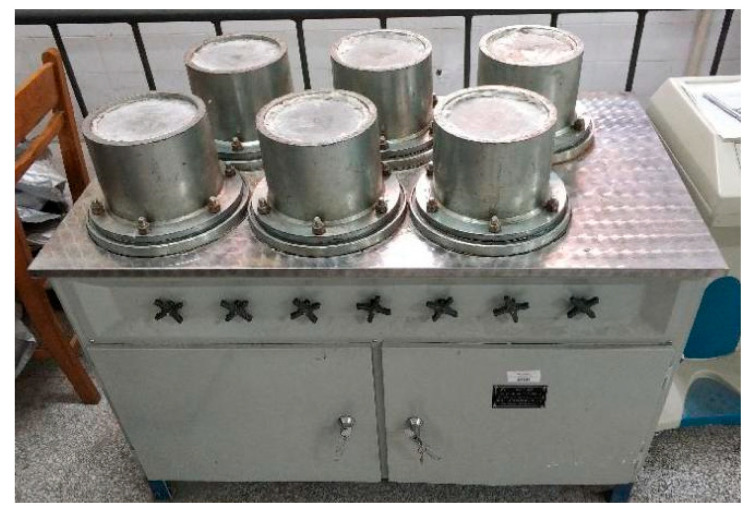
Samples installed on the impermeability instrument.

Six samples were installed on a concrete water permeability instrument, as shown in Figure 8. Because the outer surface of the sample was sealed, the water could only gradually penetrate upward from the bottom under pressure. The water pressure for the water permeability test started at 0.1 MPa and automatically increased by 0.1 MPa after every 8 h until it reached 1.2 MPa.

After the water permeability test was completed, the samples were split into two parts along the longitudinal direction by a press machine. Then the water mark of the samples after splitting was traced with a waterproof pen, and a sample was divided into 10 equal parts along the water mark with a steel ruler. The water permeability height was then measured, and the average of the 10 measured values was taken as the water permeability height. The reading was accurate to 0.1 mm. The calculation process is shown in Equation (3) [33]. The average water seepage height of each group of six samples was taken as the average water seepage height of that group of samples, as shown in Equation (4) [33].

(3) $${\bar{h}}_{i}=\frac{1}{10}\sum_{j=1}^{10}h_{j}$$

(4) $$\bar{h}=\frac{1}{6}\sum_{i=1}^{6}{\bar{h}}_{i}$$

Of which hj represents the water seepage height at the measurement point of the samples(in mm);

$\bar{h_{i}}$ represents the average water permeability height of 10 samples (mm); and

$\bar{h}$ represents the average water permeability height of a group of 6 samples (mm).

**Figure 9:**
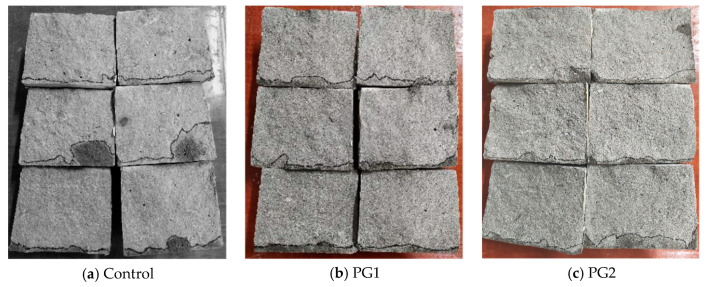
Water permeability height of three samples subjected to the water permeability test after splitting.

The water permeability height of the three samples (i.e., samples W0-1, W1-1, and W2-1) subjected to water permeability after splitting is shown in Figure 9. The area from the black line to the bottom of the samples is the water-penetrated part of the samples.

It can be seen from Figure 9 that the control sample, without PG, has the largest overall water penetrated area compared to the other two samples with PG, and the water permeability height at measurement points was uneven. Both PG1 and PG2 samples exhibited good water permeability resistance, while samples with PG1 had relatively better permeability uniformity than those with PG2. After measurement, the water permeability height of the samples at the age of 28 d was calculated and is shown in Table 3. It can be seen that the average water permeability height of the control sample without graphene was 16.7 mm, while that of the PG1 sample was 9.0 mm, which is 46.1% lower. The height of samples with PG2 was 13.0 mm, which is 22.0% lower than that of the control sample, suggesting that PG1 improved the water permeability resistance of cement-based materials more significantly than PG2.

#### 3.1.2. Chlorine Ion Permeability Test

The depth of chloride permeability of cement paste samples and related RCM test data are presented in Table 4. According to the data in Table 4 and Equation (5) [33] of the unsteady chloride ion migration coefficient, the chloride migration coefficient of the three groups of cement paste samples can be calculated as follows:

(5) $$D_{RCM}=\frac{0.0239\times (273+T)L}{(U-2)t}(X_{d}-0.0238\sqrt{\frac{(273+T){LX}_{d}}{U-2}})$$

where $D_{RCM}$ represents the unsteady chloride ion migration coefficient (as accurate as 0.1 × 10^−12^ m^2^/s);

*U* represents the absolute value of voltage used (V);

*T* represents the average of the initial temperature and end temperature of the anode solution (°C);

*L* represents the thickness of the sample (mm) (as accurate down to 0.1 mm);

*X*_d_ represents the average chloride ion permeability depth (mm) (as accurate as 0.1 mm); and

*t* represents test duration (h).

**Figure 10:**
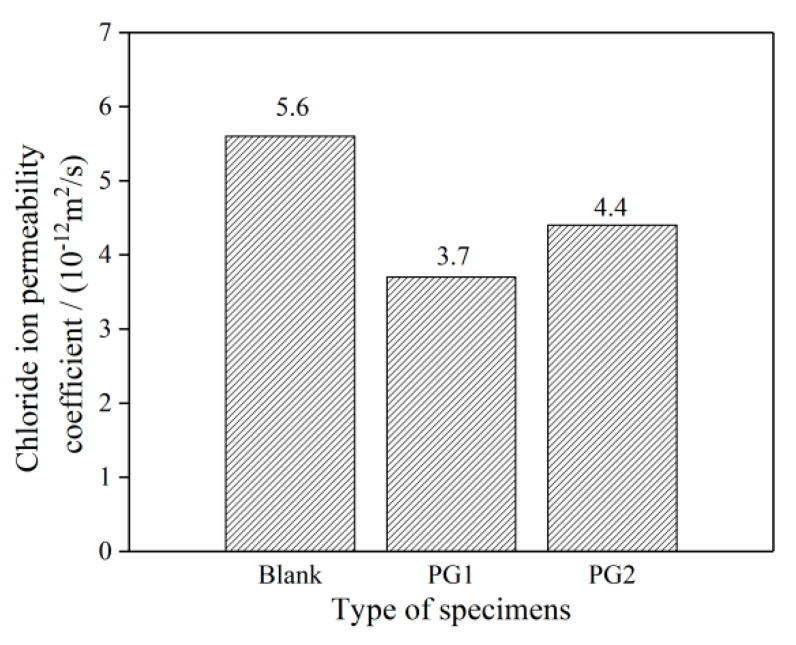
Chloride migration coefficient of the graphene-reinforced cement paste samples.

The chloride migration coefficients of the three groups of cement paste samples are shown in Figure 10. It can be seen that the addition of PG1 or PG2 can reduce the chloride migration coefficients of cement paste, and the chloride migration coefficients of cement paste with PG1 and PG2 can reach 3.7 × 10^−12^ and 4.4 × 10^−12^ m^2^/s, respectively, representing a decrease of 33.9% and 21.4%, respectively, compared to that of the control sample.

**Figure 11:**
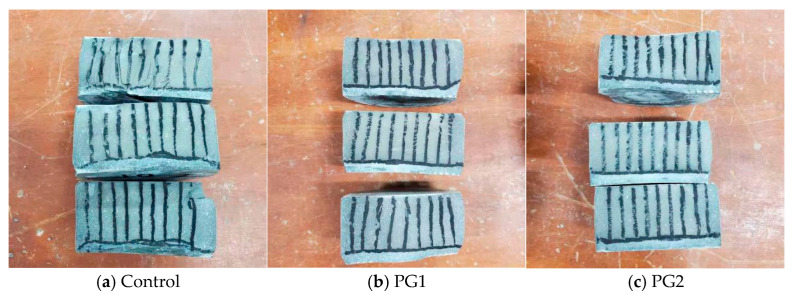
Split cement paste samples sprayed with 0.1 mol/L AgNO_3_ solution.

Figure 11 shows the split cement paste samples sprayed with AgNO_3_ solution after the permeability test, and the silver-white area that developed at the lower part of the samples was the part where chloride ions penetrated. It can be clearly seen that in the control sample, a large silvery-white area developed. However, the boundary was uneven, indicating that the internal structure of the control sample was uneven. While the silvery white area developed in the cement paste samples with PG1 and PG2 was slightly reduced compared with that in the control sample, the boundary was almost in a straight line, representing that the addition of graphene made the structure of the matrix more compact and uniform.

After adding graphene to cement samples, the water permeability resistance and chloride ion permeability resistance of cement paste samples increased significantly, which is because the two-dimensional sheet structure and the impermeable property of graphene played a bridging role in the cement matrix, thus preventing the connection of internal cracks to deflect the interconnected pores in the cement paste and prolonging the migration channel of chloride ions [34]. Due to the template effect of graphene, a large number of functional groups on its surface can provide nucleation sites for cement hydration. As a result, abundant cement hydration products can grow on the surface of graphene. Hydration products such as C-S-H and CH crystals grew divergently in a ladder-type pattern at each point. During the growth, adjacent hexagonal CH crystals collided and interlaced to affect each other [30], thus regulating the micromorphology of cement hydration products, refining grains, reducing porosity and the number of macropores, and improving compactness. 

In addition, graphene with a nanoflake structure produced a spatial crack prevention effect in the cement matrix, which can reduce and delay the generation of internal cracks in the cement matrix and thus improve the compactness of cement stones. The larger the size of graphene in a certain range, the more obvious the spatial crack prevention effect. The large-size PG1 graphene had fewer defects on the surface and more nucleation sites, whose template and bridging effects are more obvious than those with PG2. The large-size graphene is easy to curl and form a “semi-enclosed” structure. When water molecules and chloride ions penetrate the structure, they are difficult to penetrate into other parts of the cement matrix. Therefore, the hydration product structure inside the PG1 cement paste samples was denser, and both its water and chloride ion permeability resistances were higher.

### 3.2. Influence of Graphene on Water Permeability and Chloride Ion Permeability 

#### 3.2.1. XRD Phase Analysis

**Figure 12:**
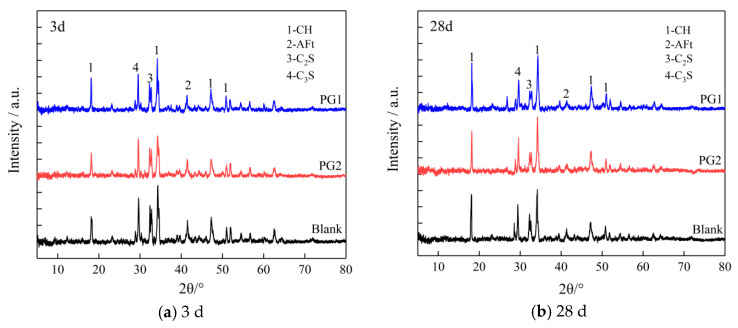
XRD patterns of cement paste samples at (**a**) 3 d and (**b**) 28 d.

Figure 12 shows the XRD patterns of the control cement slurry and PG1 and PG2 cement paste samples at 3 d and 28 d.

It can be seen that adding graphene did not change the position of the crystal diffraction peak in hydration products, suggesting that the addition of graphene did not change the type of cement hydration products but had an impact on the content and crystallinity of hydration products. Through peak intensity analysis, it is found that whether it was 3 d or 28 d, the diffraction peak of CH crystal near 18° (2θ) of the PG1 cement paste sample was significantly improved, indicating that the addition of PG1 can effectively improve the crystallization degree of CH crystal and accelerate the formation of CH crystal. This is because there are a large number of oxygen-containing functional groups on the surface and edge of PG1, providing reaction sites for the formation of CH crystals to accelerate their hydration reactions and regulate their size. This phenomenon can be explained by the classical heterogeneous nucleation theory [35]. The peak value of CH crystal diffraction of the PG2 cement paste sample had no obvious change compared with that of the control sample, indicating that the regulation effect of PG2 on CH crystal was not as obvious as that of PG1. The peak values of the AFt diffraction for PG1 and PG2 cement paste samples are lower than those of the control sample, indicating that PG1 and PG2 reduce the formation of AFt crystals. AFt crystals happen to be one of the main reasons for the occurrence of pores inside cement paste; thus, the addition of PG1 and PG2 leads to denser cement paste. The peak values of C_3_S and C_2_S diffraction for PG1 and PG2 cement paste samples are also lower than those of the control sample, indicating that the addition of PG1 and PG2 accelerates cement hydration reactions.

#### 3.2.2. SEM Micro Morphology Characterization

(1) Graphene-reinforced cement-based material samples prepared by a conventional method

**Figure 13:**
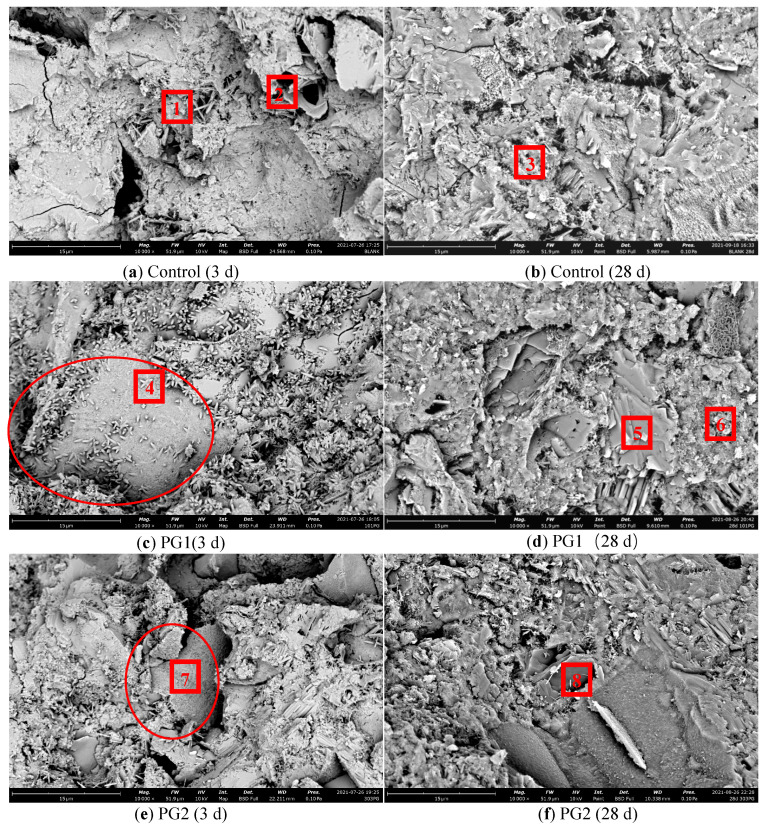
SEM images of the cement paste samples at 3 d and 28 d.

To observe the microstructure of the cement slurry sample after the incorporation of graphene, the graphene-reinforced samples and control samples without graphene were examined by SEM. The mix proportion of the samples was the same as that of the samples for the chloride ion permeability test (see Table 4), with 3 test blocks in each group. Figure 12 shows the SEM images of the samples at 3 d and 28 d. Meanwhile, to study the element types of hydration products in various samples, EDS element analysis was conducted on the eight points marked in Figure 13 during the SEM analysis, and the results are shown in Table 5.

Figure 13a,b are the SEM images of the hydration products of the control sample at 3 d and 28 d, respectively. It can be seen that there were many needle-rod and sheet hydration products in the structure, which were mainly distributed in the pores and cracks. At 28 d, a large number of hydration products were generated in the cement stone structure and grew in a staggered way. Compared with the hydration products at 3 d, its structure was more compact. However, due to the irregular shape of hydration products, there were still a large number of pores and defects in the process of growth and stacking.

Figure 13c,d are the SEM images of PG1 cement paste samples at 3 d and 28 d, respectively. Directly exposed graphene can be observed from the circle in Figure 13c. As the surface of graphene was rough, a large number of granular hydration products formed on its surface. A large number of granular hydration products were also found near point 4. These hydration products were small and closely and evenly distributed, in an outward emission shape from one point. With the progress of hydration, these granular hydration products squeezed each other [35] to effectively fill the pores in the hardened cement paste, making the structure more compact. At 28 d, the original granular hydration products continued to grow and blend, forming a dense reef-like hydration product that covered the surface of the graphene.

According to the types and data of elements in points 4 to 8 in Table 5, the content of element C at points 4, 7, and 8 increased significantly, indicating the presence of graphene.

Figure 13e,f are the SEM images of PG2 samples at 3 d and 28 d, respectively. From the data in Table 5, it can be seen that the main component of point 7 in Figure 13e was element C, indicating that the sheet material in the circle was graphene. At this time, the surface of graphene became rough and covered with a thin layer of hydration products, which were not as obvious as those in PG1. As hydration continuously progressed, hydration products around the measuring point 8 became regular reef-like ones, and the cement stone structure became denser.

One of the reasons for these phenomena is that graphene plays a bridging role [23] in the cement matrix due to its two-dimensional sheet-like structure and impermeable properties, which prevent the connection of internal cracks and extend the diffusion channels of water and chloride ions. In addition, due to the template effect of graphene, a large number of functional groups on its surface can provide nucleation sites for cement hydration, enabling significant growth of cement hydration products on the surface of the graphene, regulating the microstructure of cement hydration products, refining crystal grains, reducing porosity and the number of large pores, and improving the density of cement-based materials.

(2) Cement paste/graphene/ cement particle samples prepared by a new method

**Figure 14:**
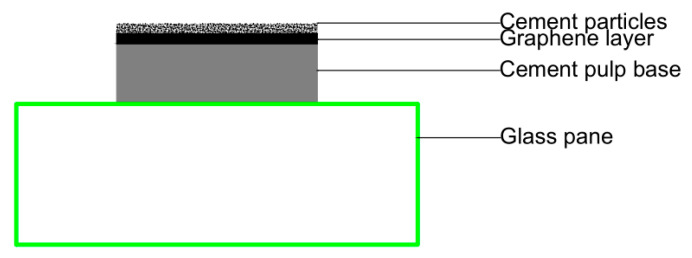
Cement paste/graphene/cement particle samples prepared by the new method.

In the above tests, graphene was distributed in a cement paste sample in a three-dimensional disordered form. It was not easy to accurately observe the interaction between cement hydration products and graphene. To further observe the hydration of cement on the surface of graphene and analyze the regulation effect of PG1 and PG2 on cement hydration products, a new method was adopted in this paper: first, two cement paste samples with a diameter of 10 mm and a thickness of about 2–3 mm were made, and after their initial setting, several drops of PG1 and PG2 graphene dispersion made by mechanical stirring and ultrasonic were respectively absorbed with a rubber head dropper and evenly coated on the surface of cement paste. The amount of graphene dispersion should just cover the surface of the cement paste samples without overflowing to make graphene nanosheets distributed on the surface of the cement paste sample. Finally, a small number of cement particles (3~5 g) were evenly sprinkled by a cement sieve with an aperture of 80 um to prepare samples of cement paste/graphene/cement particles, which were moved to an environment chamber for standard curing. The cement paste/graphene/cement particle samples prepared by this new method ensured that graphene and cement hydration products on the surface of graphene could be observed on the surface of cement paste. Its structural diagram is exhibited in Figure 14.

**Figure 15:**
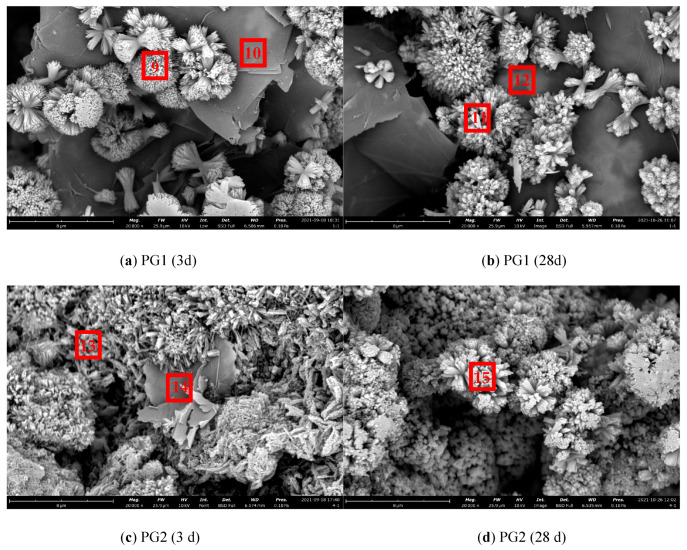
Micromorphology of cement paste/graphene/cement particle samples.

The SEM images of cement paste/graphene/cement particle specimens prepared by the above new method are shown in Figure 15, and the EDS results of corresponding measurement points are shown in Table 6.

As shown in Figure 15a, it can be observed that a large number of regular and dense flower-like cluster hydration products grew on the surface and edge of the large-diameter PG1 graphene at 3 d in an outward emission mode from one point, and most of them were concentrated at the edge. This may be due to more active sites at the edge of PG1. In Figure 15b, the hydration products increased, and more dense, flower-like clusters formed at 28 d. Due to the small amount of cement spread on graphene, the hydration products can only increase at the original reaction site and cannot be generated in large quantities, with an overall morphology similar to that at 3 d. As observed in Figure 15c, there were a large number of needle cluster hydration products at and near the edge of the smaller-diameter PG2 graphene, with shape characteristics more similar to those of traditional cement hydration products. In Figure 15d, a large number of cement hydration products were generated and covered the surface of graphene at 28 d.

From Figure 15, it can be deduced that the template effect of graphene played an important role in the cement hydration process. The functional groups on the surface of graphene can provide reaction sites for cement hydration, thus reducing the reaction barrier and improving the hydration rate. In the preparation process of graphene, the shorter the stripping and crushing process, the larger the size, the fewer the defects, and the more active oxygen-containing groups will be attached to the large-size graphene. Rather, the smaller the size, the larger the defects. Since the average sheet diameter of PG1 was larger than that of PG2, with the defects fewer and more complete, the formation of cement hydration products on the surface of graphene can be more clearly observed from PG1 samples (see Figure 15a,b).

According to the EDS element composition analysis results presented in Table 6, the content of element C at measurement points 10, 12, and 14 in Figure 14 was very high. It can be seen that PG1 graphene was at measurement points 10 and 12, PG2 graphene at measurement point 14, and cement hydration products C-S-H, Ca (OH)_2_, and a small amount of AFt were mainly at measurement points 9, 11, 13, and 15. At the same time, the content of C at those measurement points was higher than that of general cement hydration products, indicating that the cement hydration product was attached to the surface of graphene.

## 4. Conclusions

Studying the water and chloride ion permeability of cement-based materials is of great significance to assess the durability of concrete and propose measures to reduce building maintenance costs and improve building service life associated with concrete durability. Different from other studies, this paper investigated the influence of graphene on the water and chloride ion permeability of cement-based materials from the perspective of graphene size. Based on the results, the addition of either of the two types of graphene can reduce both the water and chloride ion permeability of cement-based materials. When graphene was well dispersed, large-size graphene had fewer defects and more oxygen-containing groups. Both the water and chloride ion permeability resistances of cement-based materials were improved more obviously. In this study, the cement slurry-graphene cement particle sample was prepared by a new method for the first time, and the growth law and morphology of the hydration products on the surface of graphene were observed by SEM, which further revealed the mechanism of graphene in improving the water and chloride ion permeability of cement-based materials. The specific conclusions are drawn as follows:

(1) Due to the unique nano two-dimensional sheet-like structure of graphene, which plays a bridging role and template effect in the cement matrix, it prevents the connection of internal cracks, refines the size of hydration products, improves the density of cement-based materials, and makes graphene cement-based materials more resistant to water permeability and chloride ion penetration than the blank group.
(2) XRD analysis shows that the addition of graphene does not change the type of cement hydration products but can improve the rate of the hydration reaction and accelerate the formation of CH crystals.
(3) Through scanning electron microscopy observations of graphene cement mortar specimens, it is found that during the early stage of hydration, a large number of small granular hydration products are formed on the surface of graphene, and later, regular reef-like hydration products are generated, and the structure of the cement becomes denser.
(4) By observing the newly prepared cement mortar-graphene-cement particle specimens through scanning electron microscopy, it can be directly observed that a large number of regular and dense flower clusters and needle clusters of hydration products are formed on the graphene surface. This indicates that graphene has a template effect, and its oxygen-containing groups on the surface provide reaction sites for cement hydration, regulating the morphology and size of the hydration products. As the hydration reaction proceeds, the hydration products increase, which makes the cement matrix more compact, thus improving the water permeability resistance and chloride ion permeability resistance of cement-based materials. Due to the larger average area diameter and fewer defects of PG1 than PG2, and the more complete structure, its template effect is more pronounced.
(5) Analysis of EDS elemental composition shows that the flaky substance inside the cement specimen is graphene, and the hydration products grow and cover the surface of graphene; the flower-cluster-shaped hydration products are mainly composed of C-S-H, Ca(OH)_2_, and a small amount of AFt, among others.

Because the production technology of large-diameter graphene was simpler and the cost became lower, the research methods and results proposed in this study can not only enrich the existing research on graphene cement-based materials but also provide a theoretical basis and application reference for graphene to improve the durability of cement-based materials. However, there are still some limitations. There is a lack of research on the water permeability and chloride ion permeability of graphene for cement concrete materials because, compared with cement paste, the internal structure of concrete is more complex, and the effects of different sizes of graphene on the water and chloride ion permeabilities of concrete may be different. Therefore, further research is needed in this area.

## Figures and Tables

**Table 1:** Main properties of the cement.

Product Identity	Water Demand for Normal Consistency/%	Apparent Density/(kg/m^3^)	Compressive Strength/MPa		Flexural Strength/MPa		Setting Time/min	
			3 d	28 d	3 d	28 d	Initial Setting	Final Setting
P.O 42.5	27.8	3100	≥17	≥42.5	≥3.5	≥6.5	≥45	≤600

**Table 2:** Element types and contents of the two types of graphene (wt%).

Types of GO	C	O	K	Si	Others
PG1	80.6	13.4	1.5	2.9	1.6
PG2	82.3	11.7	0.9	2.8	2.3

**Table 3:** Mix proportion of the cement samples for the water permeability test and relevant experimental results.

Code	PG1/g	PG2/g	Average Water Permeability Height of Each Group/mm	Average Total Water Permeability Height/mm
W0-1	0	0	14.1	16.7
W0-2			17.8	
W0-3			18.3	
W1-1	0.67	0	7.0	9.0
W1-2			10.4	
W1-3			9.6	
W2-1	0	0.67	12.6	13.0
W2-2			16.4	
W2-3			10.1	

**Table 4:** Mix proportions of cement samples for chloride ion permeability tests and relevant experimental results.

Code	PG1/%	PG2/%	Absolute Value of Voltage/V	Average of Initial and End Temperatures/°C	Samples’ Thickness/mm	Test Duration/h	Average Chloride Permeability Depth/mm
C0-1	0	0	20	25.5	50.1	24	6.1
C0-2			15	25.3	50.2	24	6.9
C0-3			15	25.6	50.2	24	7.0
C1-1	0.02	0	15	29.6	49.8	24	5.9
C1-2			20	29.3	51.0	24	6.0
C1-3			20	29.0	51.1	24	5.9
C2-1	0	0.02	15	29.2	51.2	24	5.9
C2-2			20	29.1	50.6	24	7.1
C2-3			20	29.1	51	24	6.6

**Table 5:** Analysis of the element types of the hydration products (% by weight).

Point	C	O	Ca	Al	Si	S	Mg	K	Fe
1	1.56	26.48	50.31	2.65	6.82	3.51	0.61	2.43	1.82
2	1.43	42.18	42.21	1.65	10.80	0.56	0.54	0.51	1.12
3	1.02	50.48	35.98	1.14	7.33	1.23	0.45	1.01	0.14
4	16.56	52.33	21.85	0.79	1.43	1.11	0.51	2.85	0.42
5	1.39	50.03	37.67	1.56	2.37	1.42	0.26	1.46	0.57
6	1.27	55.32	21.85	3.62	11.26	1.25	0.13	2.85	0.33
7	83.24	11.49	2.90	0.03	0.92	0	0	0	0
8	26.58	47.40	17.02	3.53	0.24	1.34	0.45	1.44	0.65

**Table 6:** Elemental analysis of the cement paste/graphene/cement particle samples (% by weight).

Points	C	O	Ca	Al	Si	S	Mg	K	Fe
9	24.55	49.32	17.50	1.25	3.25	0.73	0.29	1.82	0.42
10	87.84	8.75	1.26	0.42	0.27	-	-	-	-
11	12.43	43.87	30.77	1.34	2.10	1.13	0.14	0.92	0.33
12	90.13	6.21	0.89	0.32	0.18	-	-	-	-
13	24.80	47.47	16.84	1.42	1.85	1.21	0.61	1.95	1.01
14	93.24	3.51	0.23	0.10	0.04	-	-	-	-
15	11.11	47.55	28.93	2.36	6.42	0.17	0.79	1.02	0.97

## Data Availability

The authors will supply the relevant data in response to reasonable requests.
